# Factors influencing the prognosis of acute basilar artery occlusion patients treated endovascularly: the impact of treatment time window and preoperative symptoms

**DOI:** 10.3389/fneur.2023.1167442

**Published:** 2023-07-20

**Authors:** Wei Xu, Xiang Bao, Fengfeng Jiang, Feng Chen, Boxiao Liu, Fengdan Yu, Pingyou He

**Affiliations:** Department of Neurosurgery, Jinhua Central Hospital, Jinhua, Zhejiang, China

**Keywords:** basilar artery, arterial occlusive diseases, ultrasonography, interventional, risk factors, prognoses

## Abstract

**Objective:**

The aim of this study was to examine the factors influencing the prognosis of patients diagnosed with acute basilar artery occlusion (BAO) who receive endovascular treatment. Our particular emphasis was on the predictive implications of the time window for treatment (from symptom onset to femoral artery puncture) and preoperative symptoms for prognosis.

**Methods:**

A retrospective analysis of data collected from 51 BAO patients who received endovascular treatment at the Neurosurgery Department of Jinhua Central Hospital from April 2018 to October 2021 was undertaken. The data included immediate post-interventional recanalization rates and the 90-day clinical prognoses of the patients. We used the Modified Rankin Scale (mRs) to categorize patients into two prognosis groups: a favorable prognosis group (mRs score ≤2) and an unfavorable prognosis group (mRs score >2). Preoperative symptoms were gauged using the Glasgow Coma Scale (GCS) and National Institutes of Health Stroke Scale (NIHSS) scores. A logistic regression analysis was conducted to identify risk factors affecting the prognosis of BAO patients following endovascular treatment.

**Results:**

The procedure resulted in complete recanalization in all patients (100%). However, four patients (7.8%) passed away during the postoperative hospitalization period. The remaining 47 patients were followed up for 3 months. It was found that 15 patients (31.91%) had a favorable prognosis, while 32 (68.09%) had an unfavorable prognosis. It was generally observed that patients with an unfavorable prognosis had notably higher preoperative GCS and NIHSS scores (*p* < 0.05). Logistic regression analysis revealed that preoperative symptom severity, as indicated by NIHSS score, and treatment time window were significant prognostic risk factors for patients undergoing endovascular treatment for BAO (*p* < 0.05).

**Conclusion:**

Endovascular intervention for BAO appears to be safe and effective, with greater likelihood of a favorable prognosis in patients treated within ≤6 h. The chances of favorable prognosis could potentially be linked to the severity of the patient's preoperative symptoms.

## 1. Introduction

Acute basilar artery occlusion (BAO) encompasses a cluster of acute basilar artery blockages induced by acute thrombosis, dislodged emboli, atherosclerosis, or other causes. These occlusions lead to acute ischemia in crucial areas such as the cerebellum, thalamus, and brainstem, inducing corresponding neurological deficits (1, 2). BAO accounts for approximately 1% of all cases of ischemic stroke (1), and alarmingly, untreated patients have a mortality rate of 80–90% (3).

Currently, there is a lack of clinical trials affirming the safety and efficacy of endovascular interventional revascularization for BAO. In clinical stroke registry studies, it has been observed that although mechanical revascularization ameliorates revascularization rates, a favorable prognosis is achieved only in 34% of cases (4, 5). Moreover, no discernible correlation has been found between revascularization rates and prognosis (6), indicating that endovascular interventions remain ineffective for a considerable cohort of patients. The clinical outcomes of mechanical revascularization for BAO are often significantly variable, largely owing to variation in the underlying etiological mechanisms (7). Therefore, endovascular assessment of BAO is of paramount importance.

Existing research concerning prognosis in BAO has primarily concentrated on imaging characteristics, including the Alberta Stroke Program Early CT Score (ASPECTS) (8), and collateral compensation (9). Consensus has been achieved on the influence of collateral compensation on BAO prognosis (10), and this is not a subject of analysis in the present study. Despite the abundant research focusing on aspects of imaging, there remains a dearth of studies examining the implications of clinical characteristics and treatment time windows, inclusive of body temperature, on BAO prognosis. Given that preoperative clinical symptoms and the treatment time window are critical prognostic factors for endovascular interventions in patients with acute ischemic stroke (AIS) (11, 12), the current study endeavored to investigate the risk factors correlated with BAO prognosis. Specifically, we focused on the prognostic implications of the time window for treatment and of preoperative symptoms, with the objective of improving the prognosis for patients diagnosed with BAO.

## 2. Data and methods

### 2.1. Clinical data

Clinical data from a total of 51 patients with BAO who underwent endovascular treatment in the Neurosurgery Department of Jinhua Central Hospital from April 2018 to October 2021 were analyzed in this study. The inclusion criteria were: (1) diagnosis of BAO, as confirmed by magnetic resonance angiography (MRA), computed tomography angiography (CTA), or digital subtraction angiography (DSA); (2) endovascular intervention within 24 h of symptom onset; (3) age over 18 years; and (4) symptoms of neurological deficits, such as speech deficits, disorders of consciousness, limb movement dysfunction, or visual field deficits.

The exclusion criteria were: (1) cerebral hemorrhage or extensive cerebral infarction (infarct area covering more than 2/3 of the midbrain, pons, or one side of the cerebellum) as revealed by cranial CT; (2) coagulation dysfunction, indicated by a propensity for bleeding; (3) severe cardiac, pulmonary, and renal dysfunction, making the patient unable to tolerate surgery; or (4) severe disability, as determined by a modified Rankin scale (mRS) score (13) >3 before symptom onset.

Patients were evaluated, and their data were documented based on the following parameters: (i) initial assessment of patient status upon admission, determined according to the Glasgow Coma Scale (GCS) (14) and the National Institutes of Health Stroke Scale (NIHSS) (15); (ii) etiological classification, determined according to the Trial of ORG 10172 in Acute Stroke Treatment (TOAST) scheme (16); (iii) location of the arterial occlusion; (iv) administration of intravenous thrombolytic therapy before surgery; and (v) duration of the interval between onset of symptoms and femoral artery puncture, referred to as the “time window”.

### 2.2. Endovascular interventions

Following clinical assessment, a standard right femoral artery puncture was conducted under general anesthesia. After the puncture, a comprehensive brain angiogram is performed to assess the patency of specific arteries within the posterior and anterior circulation, evaluate the collateral compensation from distal blood supply to the occluded vessel, and determine the primary location of the thrombus. For vascular access, an 8F introducer catheter or a long sheath paired with an intermediate catheter was maneuvered into the vertebral artery *via* the femoral artery. To recanalize the thrombus, the aspiration catheter was navigated directly to the site of the BAO, proximal to the thrombus, where negative pressure aspiration was employed. Alternatively, a 0.014-inch microguide wire and microcatheter were utilized to traverse the thrombus, reaching the distal site of the occluded segment, under the guidance of a pathway diagram. The microguide wire was then withdrawn, awaiting the return of blood to the microcatheter before ascertaining the location of the microcatheter via hand-injected contrast medium microangiography. Provided that the microcatheter was correctly positioned within the vessel, it was then flushed with saline. Subsequently, the stent was advanced along the microcatheter to the thrombus site, and the microcatheter was withdrawn to deploy the stent. The vessel was then reimaged to verify the position of the stent and the degree of revascularization. The stent was fully deployed and left in place for 3–5 min to ensure complete embedding of the thrombus within the stent. Finally, both the stent and the microcatheter were gradually retracted outward to the exterior of the guiding catheter, where negative pressure aspiration of the intermediate catheter was applied to mitigate the likelihood of thrombus displacement, thereby reducing the re-revascularization rate.

Upon completion of revascularization, an angiogram was conducted to ascertain the antegrade flow and to quantify the procedural outcome using the modified thrombolysis in cerebral infarction (mTICI) scoring system (17). A grade of 2b/3 is defined as a successful vessel recanalization. In cases where the angiogram indicated severe stenosis (>70% stenosis) at the occlusion site, or the antegrade flow failed to reach grade 2b/3 post-revascularization, or a stable flow could not be maintained, angioplasty (comprising balloon dilation or stenting) was conducted at the stenotic site under conditions of half-volume heparinization.

Subsequent to balloon dilation, a Dyna CT scan was executed to rule out hemorrhage. Following this, tirofiban was administered at an intravenous push of 6–10 ml depending on the patient's weight, followed by a 6–8 ml/h dose *via* an intravenous pump for a duration of 24 h. Subsequently, alternating doses of aspirin (100 mg/day) and clopidogrel (75 mg/day) were administered, with tirofiban reapplication occurring after 4 h. After surgery, aspirin (100 mg/day) and clopidogrel (75 mg/day) were combined for a period of 3 to 6 months, followed by a long-term regimen of either aspirin (100 mg/day) or clopidogrel (75 mg/day), contingent upon the result of antiplatelet drug resistance testing. Perioperative and postoperative management was executed in compliance with the specifications outlined in the Chinese Guidelines for Early Endovascular Interventional Treatment of Acute Ischemic Stroke 2018 (18).

### 2.3. Postoperative data collection

An antegrade flow with an mTICI grade ranging from 0 to 2a was taken to be indicative of suboptimal revascularization, whereas flow of grade 2b or 3 was taken to suggest successful revascularization. Potential complications associated with endovascular interventions, including secondary embolism and symptomatic intracranial hemorrhage, were meticulously recorded. Patients were subjected to a follow-up period, and their mRs scores were documented 90 days post-operation. Subsequently, patients were stratified into two prognostic categories based on their 90-day postoperative mRs results. Specifically, mRs scores of 2 or less were characterized as indicative of a favorable prognosis, while mRs scores exceeding 2 were categorized indicating poor prognosis (19, 20).

### 2.4. Statistical methods

Statistical analysis was conducted using SPSS23 software (IBM). For continuous variables that conform to the normal distribution, they are expressed as mean ± standard deviation (Mean ± SD); for continuous variables that do not conform to the normal distribution, they are expressed as median and interquartile range (IQR) [M (Q1, Q3)]. Comparisons between groups were made using the Mann–Whitney U test. Categorical variables were expressed as counts and percentages, with comparisons between groups made using the Chi-square test; comparison of ranked data between the group with good prognosis and the group with poor prognosis was made using the Wilcoxon rank sum test. The independent risk factors affecting the prognosis of acute BAO patients were determined using a univariate logistic regression analysis. A P value of less than 0.05 was considered statistically significant.

## 3. Results

### 3.1. Demographic data

Demographic data were compiled for the cohort of 51 patients diagnosed with BAO. The average age among the patient population was 67 years (range: 49 to 78 years) and the sample comprised 32 men and 19 women. Some patients were identified as having habits of smoking and alcohol consumption, along with a medical history of hypertension. Further details are presented in Table 1.

**Table 1:** Demographic characteristics of 51 patients with BAO [median (IQR) or *n* (%)].

**Characteristics**	***n =* 51**
Age (years)	67 (49–78)
Gender (female)	19 (37.25)
**Lifestyle history**	
Smoking	15 (29.41)
Drinking	16 (31.37)
**Past medical history**	
Hypertension	33 (64.71)
Diabetes	16 (31.37)
Hyperlipidemia	29 (56.86)
Stroke	8 (15.69)
Cardiovascular disease	23 (45.10)

BAO, basilar artery occlusion.

### 3.2. Clinical overview of patients

**Figure 1:**
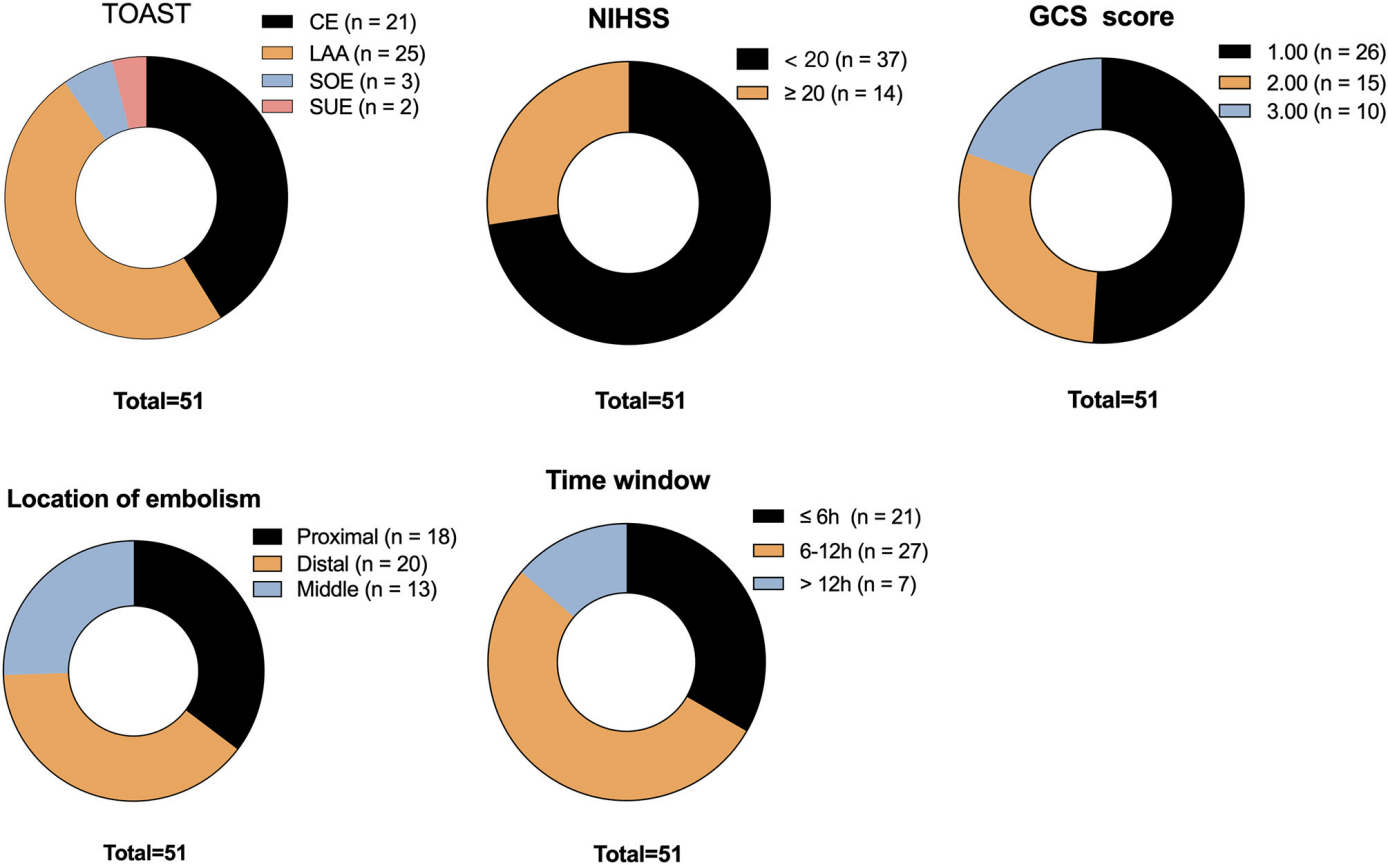
Clinical overview of 51 patients with BAO (grading and classification). CE, cardioembolism; LAA, large artery atherosclerosis; SOE, stroke of other determined etiology; SUE, stroke of undetermined etiology.

The admission and preoperative clinical parameters for the patients are summarized in Table 2. The average GCS and NIHSS scores were 9 (range: 6 to 13) and 16 (range: 12 to 21), respectively. A preponderance of the patients had experienced distal embolisms, the predominant subtype was large artery atherosclerosis (LAA), as illustrated in Figure 1.

**Table 2:** Clinical overview of 51 patients with BAO [median (IQR), *n* (%), or mean ± SD].

**Characteristics**	***n =* 51**
**Admission**	
GCS score	9 (6–13)
NIHSS score	16 (12–21)
Onset with prodromal symptoms	6 (11.76)
Temperature >38.5°C	9 (17.65)
**Preoperative**	
Time window, h	8.51 ± 3.96
Combined preoperative intravenous thrombolysis	11 (21.57)

### 3.3. Surgical results

**Figure 2:**
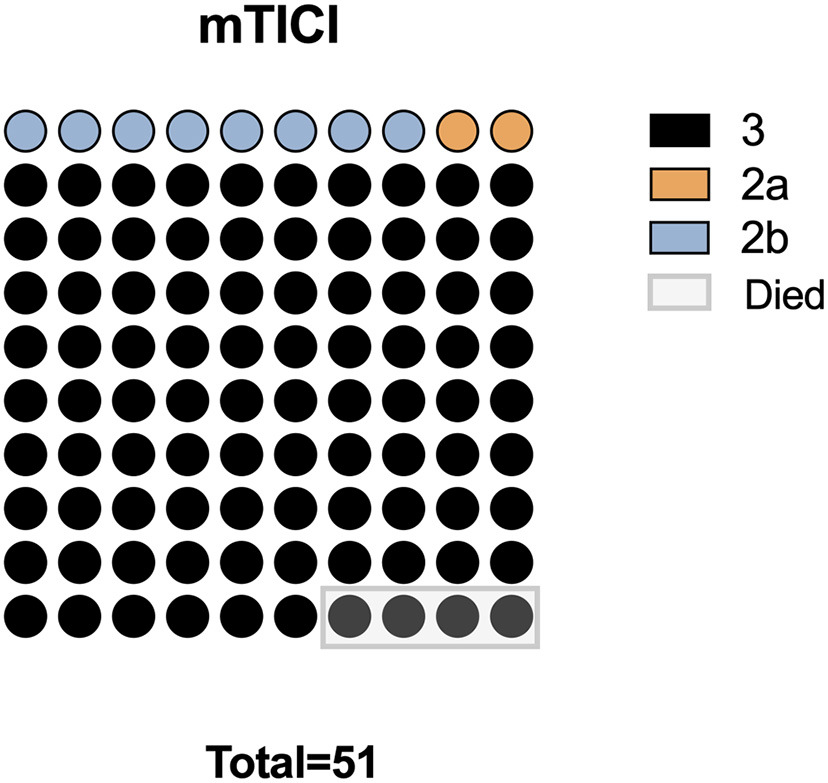
Surgical outcomes in 51 patients with BAO. mTICI, modified thrombolysis in cerebral infarction grade.

Complete recanalization was successfully accomplished in the entire patient population (100%), with 46 classified as mTICI grade 3, 4 as mTICI grade 2b, and 1 as mTICI grade 2a. During the postoperative hospitalization period, mortality occurred in four patients (7.69%); among these patients, there were two cases of postoperative central respiratory and cardiac arrest attributed to brainstem failure, one case of respiratory failure subsequent to pulmonary infection, and one case of heart failure, as illustrated in Figure 2. Additionally, symptomatic subarachnoid hemorrhage emerged in two patients (3.85%).

### 3.4. Follow-up results

Follow-up was conducted over a 3-month period for 47 patients. At 90 days post-surgery, the average recorded mRs score was 3 (range: 1 to 5), indicating a generally poor prognosis. Prognosis was defined as poor when the mRs score exceeded 2; under this scheme, 15 patients (31.91%) displayed good prognosis and 32 patients (68.09%) exhibited poor prognosis. Within this categorization, factors such as GCS score, NIHSS grade, and the location of the embolism were found to correlate with patient prognosis. Patients with a poor prognosis exhibited lower GCS scores and higher NIHSS grades, along with more frequent occurrences of embolism at the terminal and distal ends (*p* < 0.05). Details are presented in Table 3.

**Table 3:** Association between prognosis and characteristics in 47 patients with BAO [median (IQR) or *n* (%)].

**Characteristics**			**Group with good prognosis**	**Group with poor prognosis**	**U / χ^2^**	***P***
			**(mRs score** ≤**2;** ***n** =* **15)**	**(mRs score > 2;** ***n** =* **32)**		
Age (years)			67.00 (45.00–78.00)	66.00 (50.00–73.75)	227	0.773
Gender (female)			3 (20.00)	13(40.63)	1.935	0.164
**Lifestyle history**						
	Smoking		8 (53.33)	7 (21.88)	4.651	0.031
	Drinking		7 (46.67)	8 (25.00)	2.206	0.137
**Past medical history**						
	Hypertension		9 (60.00)	22 (68.75)	0.348	0.555
	Diabetes		3 (20.00)	10 (31.25)	0.646	0.422
	Hyperlipidemia		8 (53.33)	19 (59.38)	0.1525	0.696
	Stroke		4 (26.67)	4 (12.50)	1.451	0.228
	mTICI		7 (46.67)	14 (43.75)	0.035	0.851
**Admission**						
	TOAST				3.177	0.365
		CE	7 (46.67)	12 (37.50)		
		LAA	6 (40.00)	17 (53.13)		
		SOE	2 (13.33)	1 (3.15)		
		SUE	0 (0.00)	2 (6.25)		
	GCS score		12.00 (8.00–13.00)	7.00 (5.00–11.75)	140	**0.021**
	GCS grade				5.958	0.051
		3−5	0 (0.00)	10 (31.25)		
		6–8	6 (40.00)	9 (28.13)		
		9–15	9 (60.00)	13 (40.63)		
	NIHSS score		16.00 (12.00–18.00)	16.50 (13.00–26.00)	191	0.268
	NIHSS grade (score ≥ 20)		1 (6.67)	13 (40.63)	5.631	**0.018**
	Location of embolism				7.822	**0.020**
		Proximal	8 (53.33)	9 (28.13)		
		Distal	7 (46.67)	11 (34.37)		
		Middle	0 (0.00)	12 (37.50)		
	Onset with prodromal symptoms		3 (20.00)	3 (9.38)	1.035	0.309
	Temperature > 38.5°C		1 (6.67)	8 (25.00)	2.217	0.137
**Preoperative**						
	Time window		8.00 (5.50–11.00)	9.00 (7.25–10.88)	183	0.196
	Time window classification				2.279	0.320
		> 12 h	1 (6.67)	6 (18.75)		
		6–12 h	8 (53.33)	19 (59.38)		
		≤6 h	6 (40.00)	7 (21.87)		
	Intravenous thrombolysis		2 (13.33)	5 (15.62)	0.042	0.837
**Postoperative**						
	mTICI grade				2.623	0.269
		2a	0 (0.00)	1 (3.15)		
		2b	0 (0.00)	4 (12.50)		
		3	15 (100.00)	27 (84.35)		

Bolded values represent *P* < 0.05, a significant difference.

### 3.5. Logistic regression analysis

Upon deeper analysis of the cohort data, and after controlling for variables such as time window, GCS score, and NIHSS score, it was determined that patients who presented with higher NIHSS scores and whose treatment time window exceeded 6 h experienced significantly worse prognosis compared to those with lower NIHSS scores (below 20) and shorter treatment time windows (≤6 h). This increment in risk was statistically significant (*P* < 0.05), as outlined in Table 4.

**Table 4:** Logistic regression analysis for prognosis over 47 patients.

**Variables**		**B**	**S.E**.	**Wald**	**df**	**OR**	**95% CI for OR**		***P***

							**Lower**	**Upper**	
NIHSS score	<20					1.000			**0.012**
	≥20	3.624	1.445	6.290	1.000	37.503	2.208	637.047	
Time window	≤6 h					1.000			**0.034**
	>6h	2.431	1.144	4.512	1.000	11.368	1.207	107.096	

Bolded values represent *P* < 0.05, which is a significant difference.

## 4. Discussion

Schonewille et al. (21) documented a favorable prognosis rate of under 20% and a mortality rate reaching 40% in cases of BAO treated via conventional methods. Endovascular treatment, in comparison to the conventional approach, offers the advantage of swiftly unblocking the occluded vessels to achieve reperfusion, thereby salvaging as much ischemic hemispheric brain tissue as possible, which in turn improves clinical outcomes. The primary complication associated with this procedure is intracranial hemorrhage. Ravindren et al. (21) reported that, among 231 patients suffering from acute BAO who were treated *via* endovascular procedures, intracranial hemorrhage was observed in more than 14% of cases. The degree of safety and efficacy of endovascular intervention in patients experiencing acute BAO remains uncertain (22).

In the current study, both instances of symptomatic hemorrhage were subarachnoid hemorrhages. These resulted from the displacement of a basilar artery thrombus into the P2 segment of the posterior cerebral artery with a convoluted vessel and the subsequent stenting of the P2 segment, leading to straightening of the vessel to the point of inducing endothelial injury. The symptomatic cerebral hemorrhage rate in patients treated with endovascular therapy for acute BAO has been previously reported in the literature to range between 1.9 and 6% (22, 23). In the present study, among 51 patients, there were two instances of hemorrhage, for a hemorrhage rate of 3.9%. This relatively low hemorrhage rate can be attributed to the fact that recanalization of the occluded vessel was achieved through direct aspiration in nearly half of the cases. Machado et al. (24) reported a favorable prognosis rate of 43.5% at 90 days post-endovascular treatment of acute BAO. Similarly, in an international multicenter registry study that included 148 patients with BAO who underwent endovascular treatment, 79% achieved revascularization, and 34% achieved a favorable prognosis (4).

In the present study, the observed rate of favorable prognosis in cases of BAO treated with endovascular intervention was 37.2%, and the mortality rate stood at 15.6%. These rates are generally aligned with the latter report mentioned, and the mortality rate of patients with acute BAO treated via endovascular therapy was notably lower than that associated with conventional pharmacotherapy. A comprehensive analysis of 20-year prognosis and mortality data from 207 BAO cases from a single large center reported superior rates of favorable and intermediate prognosis and extended life expectancy among survivors post-endovascular treatment (25). These findings suggest, to a certain extent, that endovascular interventions may considerably enhance the rate of favorable prognosis and mitigate mortality in patients, affirming their safety and effectiveness. Additionally, preoperative clinical symptoms have been reported in the literature as a factor impacting the outcome of endovascular interventions in patients with acute BAO: the more severe the preoperative symptoms, the poorer the prognosis. In the present study, an NIHSS score of ≥20 was associated with poor clinical outcomes, and multifactorial analysis demonstrated that an NIHSS score of ≥20 was a significant contributor to poor prognosis (*P* = 0.012). This suggests that even if complete revascularization of a patient's occluded vessel is achieved through endovascular treatment, poor prognosis is still likely, with a high rate of disability and mortality.

The severity of preoperative clinical symptoms was also thoroughly evaluated using the GCS score, whereby scores of 9–15 (observed in 26 cases) were classified as mild to moderate, scores of 6–8 (seen in 15 cases) were deemed severe, and scores of 3–5 (noted in 10 cases) were categorized as extra severe. For the 10 patients assessed as having extra-severe symptoms, with a GCS score of 3–5, the rate of favorable prognosis was 0, suggesting that patients presenting with a preoperative GCS score of 3–5 are more likely to encounter a poor prognosis. In their investigation of endovascular treatment outcomes for BAO patients, Yue et al. (26) identified preoperative symptom severity as an independent risk factor influencing prognosis (26), noting that patients with extra-severe preoperative symptoms had poorer prognosis, a finding generally congruent with the above reports. The correlation between the therapeutic time window and prognosis for patients receiving endovascular treatment for BAO is not yet fully understood. A multicenter retrospective study comprising 212 BAO patients revealed no statistically significant difference between the favorable and poor prognosis groups in terms of time elapsed from symptom onset to vessel recanalization (27). The retrospective literature supports the effectiveness of an intervention window of over 6 h for endovascular treatment of BAO (28). Singer et al. reported a 34% rate of favorable prognosis at 3 months and a mortality rate of 35% among 148 patients with BAO who underwent endovascular treatment, identifying 9 h as a crucial intervention window, with poor prognosis rates increasing after this time (4). Mokin et al. (5) noted a favorable prognosis rate for patients treated within a 6-h window that was double that among patients treated beyond 6 h after symptom onset. A prospective multicenter study reported that an extended interval between symptom onset and vessel recanalization in acute BAO patients was correlated with increased rates of poor prognosis, morbidity, and mortality, with intervals exceeding 6 h being associated with poor prognosis (29). Sang et al. (30) reported enhanced outcomes in cases in which endovascular therapy was initiated within the first 9 h post-onset in patients with acute BAO, but suggested that the benefit may remain consistent thereafter, implying that early endovascular intervention correlates with improved prognosis. The univariate analysis conducted in this study indicated no statistically significant differences in prognosis between patients treated within the interval ranges of ≤6 h, 6–12 h, and >12 h (*P* = 0.32). However, considering the small sample size of this study, the results pointed to a higher rate of favorable prognosis in cases of treatment within a <6 h window. Consequently, the time window may be a crucial factor in predicting prognosis for patients with acute BAO, although further validation with a larger sample size remains necessary.

This study does bear certain limitations. As a retrospective investigation with a limited sample size, the findings may be subject to potential bias. Further validation through a multicenter, large-sample, prospective, randomized controlled study would be advantageous in order to delve deeper into the safety and efficacy of endovascular treatment for BAO and the factors influencing prognosis in patients receiving this treatment.

## 5. Conclusion

In conclusion, endovascular interventions are safe and effective in patients with BAO and can be considered for further expansion. Patients who are treated within a short time window and who have milder preoperative symptoms achieve a good prognosis at higher rates, and effective endovascular interventions can improve the prognosis of patients when carried out as soon as possible.

## Data availability statement

The raw data supporting the conclusions of this article will be made available by the authors, without undue reservation.

## Ethics statement

The studies involving human participants were reviewed and approved by the Ethics Committee of Jinhua Central Hospital. The patients/participants provided their written informed consent to participate in this study.

## Author contributions

WX contributed to the concept and design of the study. XB and FJ performed the statistical analysis. FC, BL, FY, and PH wrote parts of the manuscript. All authors were involved in revising, reading, and approving the submitted version of the manuscript.
